# Long Non-coding RNA SENP3-EIF4A1 Functions as a Sponge of miR-195-5p to Drive Triple-Negative Breast Cancer Progress by Overexpressing CCNE1

**DOI:** 10.3389/fcell.2021.647527

**Published:** 2021-03-15

**Authors:** Lie Chen, Xiaofei Miao, Chenchen Si, An Qin, Ye Zhang, Chunqiang Chu, Zengyao Li, Tong Wang, Xiao Liu

**Affiliations:** ^1^Department of Thyroid and Breast Surgery, Wuxi People's Hospital Affiliated to Nanjing Medical University, Wuxi, China; ^2^Department of General Surgery, Wuxi People's Hospital Affiliated to Nanjing Medical University, Wuxi, China; ^3^Dermatological Department, Wuxi Children's Hospital Affiliated to Nanjing Medical University, Wuxi, China

**Keywords:** triple-negative breast cancer, CCNE1, microRNA, LncRNA, DNA methylation

## Abstract

Triple-negative breast cancer (TNBC) has high malignancy and limited treatment, so novel molecular therapeutic targets are urgently needed. Cyclin E1 (CCNE1) promotes progression in breast cancer, but its role and inherent mechanisms in TNBC are yet to be elucidated. Competing endogenous RNA (ceRNA) may be a potential mechanism. CCNE1 was selected though bioinformatics and clinical samples, and cell lines were utilized to verify CCNE1 expression by qRT-PCR and western blot. Predicting tools provided potential miR-195-5p and SENP3-EIF4A1 and tested from multilevel. Functional experiments were conducted *in vitro* and *in vivo*. Luciferase reporter assay and RNA immunoprecipitation experiments were implemented to ensure the interaction between miR-195-5p and SENP3-EIF4A1/CCNE1 in TNBC. Bioinformatics found DNA hypermethylation of miR-195-5p and preliminarily verified. Mechanistically, SENP3-EIF4A1-miR-195-5p-associated ceRNA could drive TNBC progress though regulating CCNE1. DNA hypermethylation of miR-195-5p might be another reason. In summary, SENP3-EIF4A1-miR-195-5p-CCNE1 axis promotes TNBC progress and may contribute to the novel diagnosis and treatment of TNBC.

## Introduction

According to the 2020 cancer statistics published by the American Cancer Society (ACS) in CA, 276,480 cases of breast cancer are estimated to be the first in the incidence rate of female malignant tumors (Siegel et al., 2020). Breast cancer is one of the most common malignant tumors in women, which should not be ignored. Triple-negative breast cancer (TNBC), accounting for about 15–20% of total, is a subtype characterized by lack of estrogen receptor (ER) and progesterone receptor (PR) expression and lack of ERBB2 (also known as HER2) amplification (Foulkes et al., 2010). More than 75% have features of Basal-like subtype (Perou et al., 2000); TNBC always show more aggressive clinical behaviors (Sorlie et al., 2001) and poor prognosis (Carey et al., 2006). Due to the lack of effective therapeutic targets, the treatment of TNBC patients is extremely limited; the treatment bottleneck is difficult to break through. Thus, TNBC patients need new treatment options, and discovery of novel molecular targets is the priority.

Cyclins are widely distributed in eukaryotes from yeast to human. They participate in cell cycle regulation by interacting with cyclin-dependent kinases (CDKs) and cyclin-dependent kinase inhibitors (CKIs). Cyclin E1 (CCNE1) is one of the subtypes of cyclin E, which is considered as G1-cyclin. CCNE1-CKD2 complex induces hyperphosphorylation of phosphorylated Rb protein initiated by cyclin D-CDK4/6 (Macaluso et al., 2006), releasing E2F, allowing cell cycle from G1 to S phase and DNA synthesis (Harbour et al., 1999; Caldon and Musgrove, 2010). A number of studies have shown that CCNE1 is abnormally expressed in a variety of malignant tumors (Au-Yeung et al., 2017; Aziz et al., 2019). For breast cancer, overexpression of CCNE1 have been verified as an early event of breast cancer progression (Shaye et al., 2009), and high level of CCNE1 is often associated with poor prognosis (Fredholm et al., 2017). However, the expression and function of CCNE1 in TNBC is rarely studied.

At present, the theory of competitive endogenous RNA (ceRNA) hypothesis is a hot topic in oncology (Zheng et al., 2018; Wang W. et al., 2020). MicroRNA, long non-coding RNA (LncRNA), mRNA, and pseudogenes can interact and regulate each other through competitive binding microRNA response element (MRE) (Salmena et al., 2011), which provides a new gene regulation mechanism. miRNA is a non-encoding RNA of 18–25 nucleotides, which function is as a posttranscriptional regulator of target mRNA (Garzon et al., 2010; Kasinski and Slack, 2011). Recent studies have shown that miRNA can be involved in the occurrence and progression of many malignant tumors, including breast cancer or even TNBC (Givel et al., 2018; Kong et al., 2018). LncRNA plays an important role in ceRNA hypothesis. LncRNA is ubiquitous in the human genome (Jarroux et al., 2017) and is indispensable for maintaining the growth and function of normal cells (Jathar et al., 2017). The anomalous expression of lncRNA is closely related to the occurrence and development of many malignant tumors, including breast cancer (Wang et al., 2019; Yao et al., 2019). Although lncRNA-microRNA-associated ceRNA has been confirmed to regulate CCNE1 expression in hepatocellular carcinoma (Li et al., 2019), it is still unclear if this could affect TNBC.

Our study aims to investigate the relationship between lncRNA-microRNA-associated ceRNA and CCNE1 in patients with TNBC. The results clarify that lncRNA SENP3-EIF4A1 and miR-195-5p act on CCNE1 and regulate its promotion of TNBC progress.

## Materials and Methods

### Cell Lines and Clinical Samples

MCF-7, MDA-MB-231, T47D, and ZR-75-1 cell lines were purchased from the American Tissue Culture Collection (ATCC). HBL-100 cell line came from Shanghai Institute of Biological Sciences. While, SUM1315 cell line was provided by the University of Michigan. DMEM (Gbico, Detroit, MI, USA) and 10% FBS (Gbico, Detroit, MI, USA), combined with 1% penicillin/streptomycin (Gibco, Detroit, MI, USA), were utilized to culture MCF-7, MDA-MB-231, and SUM1315. Differently, HBL-100, T47D, and ZR-75-1 cell lines were grown and reproduced in RPMI1640 (Gbico, Detroit, MI, USA) mixed with the same FBS and antibiotics. Forty-five paired breast cancer tissues (30 TNBC) and adjacent normal tissues were gathered from breast cancer patients who underwent surgical treatment at Nanjing Medical University Affiliated Wuxi People Hospital (Wuxi, China). Preoperative treatment was not performed. The study was approved by the Nanjing Medical University Institutional Ethics Committee and obtained written consent from the participants.

### Lentivirus, Plasmid, and Small Interfering RNA Transfection

The miR-195-5p mimics/inhibitor lentivirus and their negative control were designed by GenePharma (Shanghai, China). Both two TNBC cell lines MDA-MB-231 and SUM1315 were transfected with miR-mimics/inhibitor or negative control when cells are at ~50% confluence. Three micrograms per milliliter of puromycin (VWR, USA) was used to select transfected cells for about 2 weeks.

Lipofectamine 3000 (Invitrogen, Carlsbad, CA, USA) was used as a vector to transfect SENP3-EIF4A1 plasmids and small interfering RNAs (siRNAs) (GenePharma) into chosen breast cancer cells.

### Quantitative Real-Time PCR and Western Blot

The total RNA was extracted with RNAiso Plus (Takara, Kusatsu, Japan) from collected clinical samples and chosen breast cancer cells, according to the manufacturer's protocol. cDNA was specifically synthesized for miRNA, and then was tested using the Hairpin-it miRNA qPCR Quantitation Kit (GenePharma, China). Other cDNA was synthesized by the reverse transcription kit (Takara), and then detected by the SYBR Green Master Mix Kit (Roche, Reinach, Switzerland). The expressions were normalized based on U6 or GAPDH, respectively. The primers for targets are listed in Supplementary Table 1.

The primary antibodies utilized for western blot were as follows: anti-CCNE1 (Abcam, Cambridge, UK, 1:1,000, ab33911), anti-CDK2 (Abcam, 1:3,000, ab32174), anti-c-Myc (Abcam, 1:1,000, ab32072), anti-E2F1 (Abcam, 1:1,000, ab 112580), and anti-GAPDH (Abcam, 1:2,500, ab9485). The secondary antibodies (GOAT anti-mouse and anti-rabbit IgG) were from Jackson Immunoresearch (USA).

### RNA Immunoprecipitation

RNA immunoprecipitation (RIP) was performed by the Magna RIP kit (Millipore, Billerica, USA) according to the manufacturer's requirement. SUM1315 cells transfected miR-195-5p mimics/NC were cultured for 2 days and then lysed. After mixture with anti-Argonaute2 (AGO2) or IgG (negative control) conjugated magnetic beads for 4 h at 4°C, the mixture was washed. Then, purified and enriched microRNAs and lncRNA were detected using quantitative real-time PCR (qRT-PCR) and agarose gel electrophoresis.

### Dual-Luciferase Reporter Assay

The wild-type (WT) or mutant (Mut) miR-195-5p binding site in the CCNE1 and SENP3-EIF4A1 were designed and constructed from GenePharma (China). After being transfected and cultured for 2 days, the Firefly and Renilla luciferase activities were tested by the Dual Luciferase Reporter Assay Kit (Promega, WI, USA, E1910).

### Cell Proliferation and Cell Cycle Assays

According to the manufacturer's instructions, we applied the cell counting kit-8 (CCK8, Beyotime, Shanghai, China), clone formation, and 5-ethynyl-2′-deoxyuridine (EdU) kit (RiboBio, Guangzhou, China) to demonstrate the growth ability of TNBC.

For cell cycle analysis, chosen cells were collected and washed, then fixed in 70% ethanol at −20°C overnight before FCM. Cell cycle analysis kit (MultiSciences, Hangzhou, China) was used to dye for 15 min. Data were analyzed by a FACScan flow cytometer.

### 5-Aza-2′-Deoxycytidine Application

5-Aza-2′-deoxycytidine (5-AzaDC) (MCE, USA) with concentration gradient was added into MDA-MB-231 cells at about 50% confluence and cultured for 3 days. We collected related RNA and protein for qRT-PCR and western blot.

### Animal Experiments

Animal experiments followed the protocols stipulated by the Nanjing Medical University Institutional Animal Care and Use Committee. Five-week-old female Balb/c nude mice from the animal center of Nanjing Medical University were used to construct xenotransplantation model. About 2 × 10^6^ SUM1315 cells which stably transfected miR-mimics/inhibitor and negative controls were injected subcutaneously into the blank area of the forelimb. Tumors were gauged every 4 days after the constructed and the experimental mice were benevolently killed 28 days after injection. Tumor volume = (width^2^ × length)/2. Survival analysis started at day 24.

### Bioinformatics Analysis

The Cancer Genome Atlas (TCGA) database (https://portal.gdc.cancer.gov), including RNA expression, methylation level, and clinical imformation of breast cancer samples, was ustilized in this study. TCGA data was downloaded from the TCGA database under the accession numbers: TCGA BRCA. GTEx (https://gtexportal.org/) database, which increased the number of normal samples, was applied to perform a pan-cancer analysis of CCNE1, and cooperated with TCGA database.

MIRWALK (http://mirwalk.umm.uni-heidelberg.de/), MIRTARBASE (http:// mirtarbase.cuhk.edu.cn/php/index.php), and MIRDIP (http://ophid.utoronto.ca/ mirDIP/ index. Jsp) are three frequently used mircoRNA-related forecasting tools and were applied jointly to predict potential mircoRNA or lncRNA.

### Statistical Analysis

All experiments were performed at least three times with samples in triplicates. We used SPSS 20.0 (Chicago, IL, USA) and Graphpad Prism 8.0 (California, China) to perform statistical analysis. The ANOVA with a *post-hoc* test (Dunnett's multiple comparisons test) and two-tailed Student's *t*-test were utilized for studies with more than two groups and only two groups, respectively. Besides, the Pearson correlation was applied to analyze the correlation of expression between two different factors. *p* < 0.05 indicated a statistically significant difference. ^*^p < 0.05; ^**^p < 0.01; ^***^p < 0.001, and ^****^p < 0.0001.

## Results

### CCNE1 Expression Profiles in TNBC

**Figure 1:**
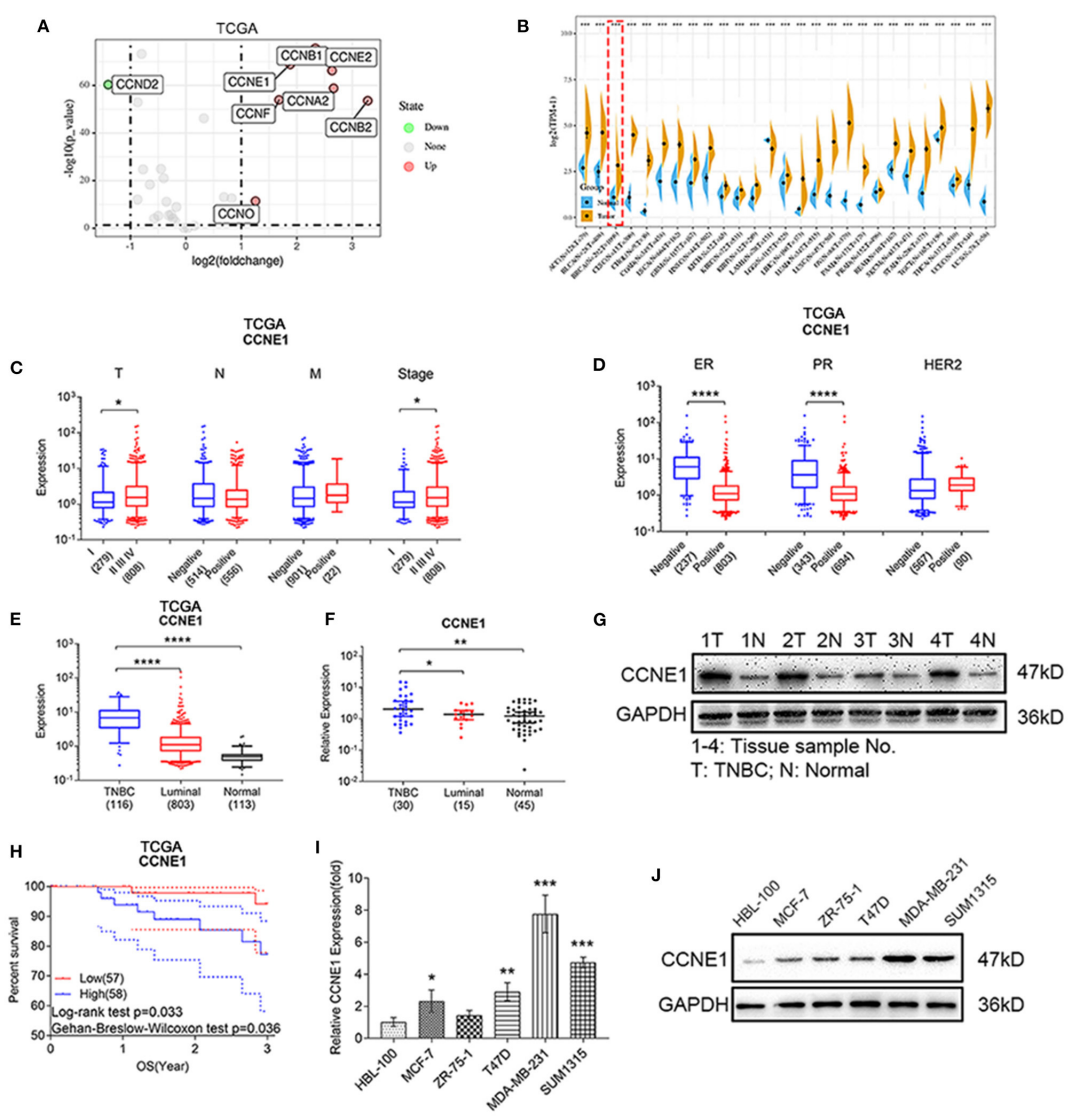
CCNE1 are overexpressed in TNBC and associated with poor prognosis. **(A)** Expression difference analysis of cyclins according to TCGA BRCA database [log2(fold change) >1, *p* < 0.05]. **(B)** Pan-cancer analysis of CCNE1 by TCGA and GTEx database. **(C,D)** CCNE1 expression in different TNM stages **(C)** or ER/PR/HER-2 status **(D)** in TCGA BRCA database. **(E)** CCNE1 expression in TNBC samples as compared with luminal-type breast cancer or normal breast samples from TCGA database. **(F,G)** CCNE1 expression in 30 human TNBC or 15 luminal-type breast cancer tissues and 45 adjacent normal breast tissues were detected by qRT-PCR **(F)** and four pairs by western blot **(G)**. 1–4, tissue sample No.; T, TNBC tissue; N, normal breast tissue. **(H)** Survival curve of 3-year overall survival in 115 patients with TNBC according to the CCNE1 expression from TCGA database. **(I,J)** Expression of CCNE1 in breast cancer cell lines and HBL-100 were detected by qRT-PCR **(I)** and western blot **(J)**. Data are presented as mean ± SD. *p < 0.05; **p < 0.01; ***p < 0.001; ****p < 0.0001.

In order to understand initially cyclin expression in breast cancer, we utilized TCGA BRCA database to perform expression difference analysis. We found that eight cyclins (CCNA2, CCNB1, CCNB2, CCND2, CCNE1, CCNE2, CCNF, CCNO) were differentially expressed between breast cancer and normal tissue samples (Figure 1A), with the cut-off criterion of log2(fold change) >1, *p* < 0.05. This result showed that CCNE1 was significantly upregulated (3.68-fold) in breast cancer. Then, TCGA database and GTEx database were applied synergistically to design pan-cancer analysis. CCNE1 was overexpressed in several types of cancer, including breast cancer (Figure 1B). Combined TCGA BRCA clinical database, we found that breast cancer samples with higher level of T stage or TNM stage show higher expression of CCNE1 (Figure 1C). At the same time, ER or PR samples expressed high level of CCNE1 (Figure 1D).

Analyzing the TCGA BRCA database, CCNE1 was found to be the highest expression in TNBC compared with luminal-type breast cancer and normal samples (Figure 1E; Supplementary Figure 1A). Moreover, high-level expression of CCNE1 in TNBC showed bad overall survival (OS) (Figure 1H). qRT-PCR were performed to test CCNE1 mRNA expression in collected clinic samples of TNBC, luminal-type breast cancer, and corresponding normal tissues and CCNE1 expressed highest level in TNBC (Figure 1F; Supplementary Figure 1B). Whereas, results of western blot showed that CCNE1 expression was also higher in TNBC (Figure 1G). For breast cancer cell lines, qRT-PCR and western blot analysis confirmed that CCNE1 expression was higher in MDA-MB-231 and SUM1315 (two TNBC cell lines) than other types of breast cancer cell lines (MCF-7, ZR-75-1, and T47D) and normal breast epithelial cell line (HBL-100) (Figures 1I,J).

Hence, we paid our attention to CCNE1 in tumorigenesis and development of TNBC in this research.

### miR-195-5p Expression Profiles in TNBC

**Figure 2:**
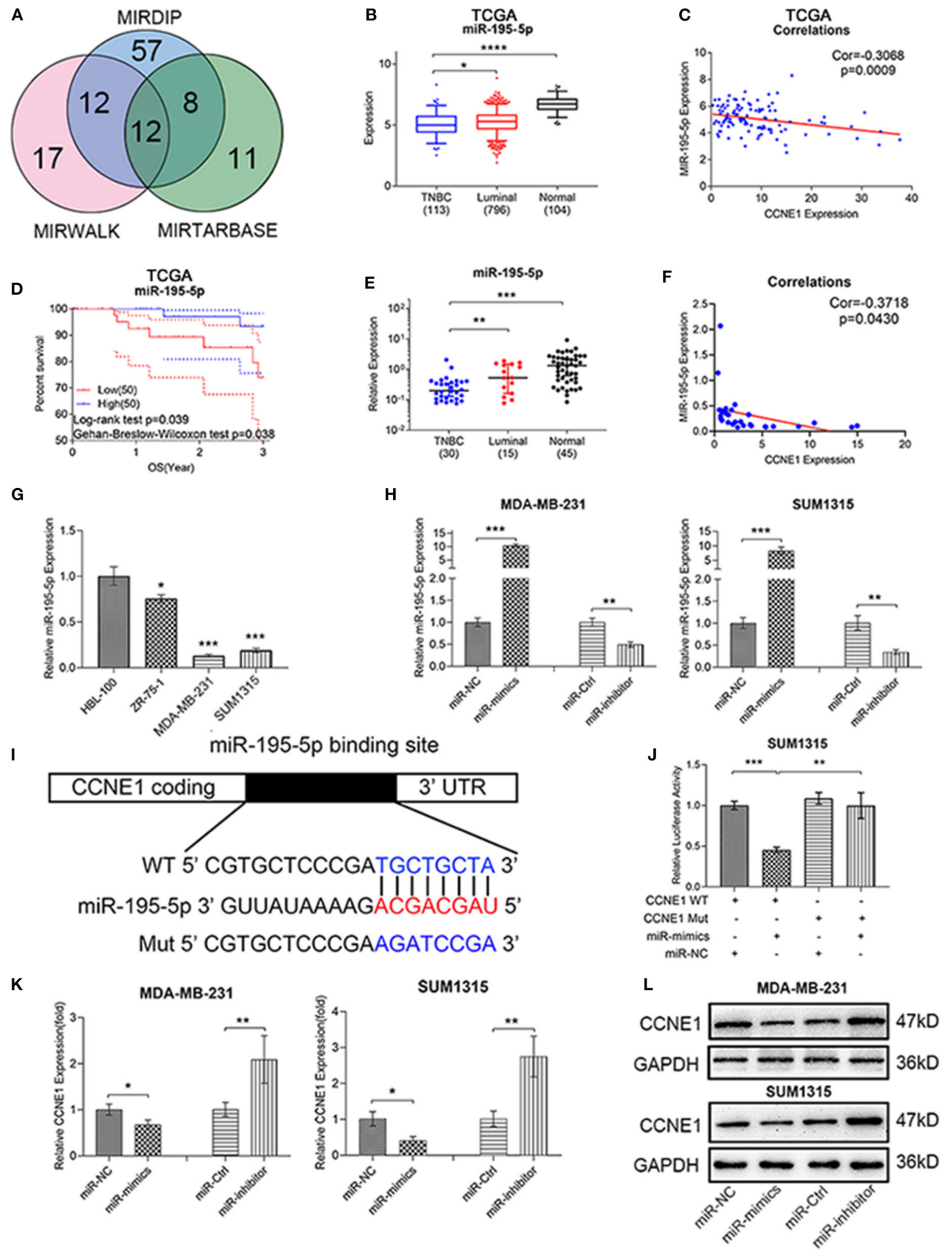
miR-195-5p acts as a negative regulator for CCNE1 in TNBC. **(A)** MIRWALK, MIRTARBASE, and MIRDIP predicted potentially related microRNAs of CCNE1. **(B–D)** TCGA BRCA database showed miR-195-5p expression in TNBC samples as compared with luminal-type breast cancer or normal breast samples **(B)**, miR-195-5p and CCNE1 expression correlation in TNBC (*n* = 113) **(C)**, and 3-year overall survival in 100 patients with TNBC according to the miR-195-5p expression **(D)**. **(E,F)** miR-195-5p expression in 30 human TNBC or 15 luminal-type breast cancer tissues and 45 adjacent normal breast tissues **(E)** and expression correlation with CCNE1 in 30 TNBC samples **(F)**. **(G)** Expression of miR-195-5p in breast cancer cell lines and HBL-100. **(H)** Relative RNA levels of miR-195-5p were detected in cells after being transfected with miR-NC, miR-mimics, miR-Ctrl, and miR-inhibitor using qRT-PCR. **(I)** Schematic illustration of CCNE1 luciferase reporter vectors. **(J)** The relative luciferase activities were detected in SUM1315 cells after transfection. **(K,L)** Relative mRNA **(K)** and protein levels **(L)** of CCNE1 were detected in cells after being transfected with miR-NC, miR-mimics, miR-Ctrl, and miR-inhibitor. Data are presented as mean ± SD. *p < 0.05; **p < 0.01; ***p < 0.001; ****p < 0.0001.

To predict CCNE1-related miRNA, three miRNA target prediction databases online (MIRWALK, MIRTARBASE, and MIRDIP) were applied. Venn diagram showed 12 potential miRNAs (Figure 2A), including miR-195-5p. We further found that miR-195-5p expression was lower in TNBC than other groups of samples (Figure 2B; Supplementary Figure 1C) and high miR-195-5p level in TNBC showed good OS (Figure 2D). Clinical samples and breast cancer cell lines verified above result (Figures 2E,G; Supplementary Figure 1D).

In order to ensure the relationship of miR-195-5p and CCNE1, Pearson correlation analysis was performed. These results indicated that miR-195-5p expression was negatively associated with CCNE1 expression in TNBC, whether it was TCGA or clinical data (Figures 2C,F). Transfecting miR-195-5p mimics or inhibitor to MDA-MB-231 and SUM1315 cell lines may upregulate or downregulate miR-195-5p expression in these two cell lines, respectively. Effects were verified by qRT-PCR (Figure 2H). Then we applied the dual-luciferase reporter analysis to identify that CCNE1 was a direct target of miR-195-5p. Fragments were performed (Figure 2I). The luciferase activity of miR-195-5p was decreased in SUM1315 cells with wild-type CCNE1 3′UTR, while the activity was not different in mutated CCNE1 3′UTR (Figure 2J). At the same time, miR-195-5p inhibitor could markedly upregulate CCNE1 mRNA expression, whereas mimics could significantly downregulate the level both in MDA-MB-231 and SUM1315 cell lines (Figure 2K). As shown in Figure 2L, the protein expression levels of CCNE1 were also altered by miR-195-5p inhibitor or mimics in two TNBC cell lines (Figure 2L).

These results ensured that miR-195-5p was the potential up-stream factor of CCNE1 and miR-195-5p might take participation in tumorigenesis and progression of TNBC.

### The Inhibitory Effect of miR-195-5p in TNBC Proliferation

**Figure 3:**
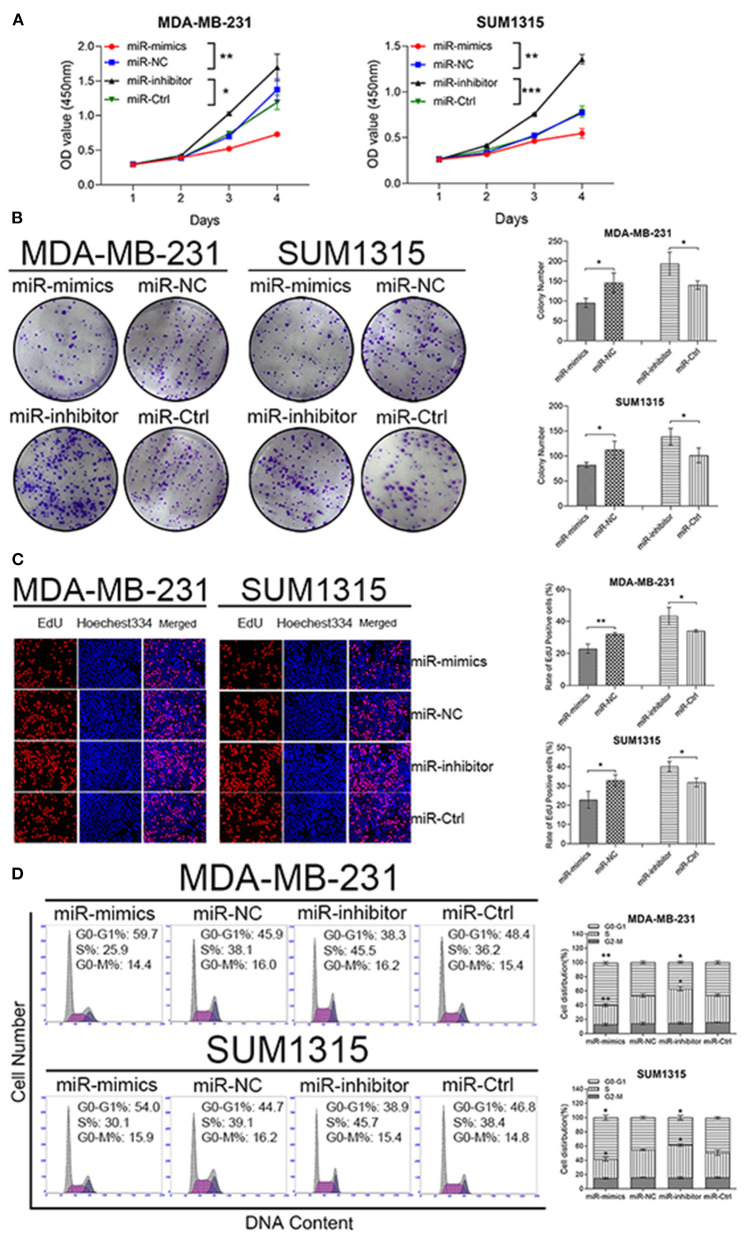
miR-195-5p promotes TNBC cell proliferation and modulates cell cycle. **(A)** The growth curves of transfected cells were evaluated by CCK8 assays. **(B)** Colony formation assays were used to detect the growth of transfected cells. **(C)** EdU assays were performed in transfected cells. **(D)** Flow cytometry assay analyze cell cycle of transfected cells. Data were presented as mean ± SD. *p < 0.05; **p < 0.01; ***p < 0.001.

To reveal biological functions of miR-195-5p in TNBC, CCK8 assay-related growth curves and colony formation assays were performed. Both results demonstrated that upregulation of miR-195-5p could reduce proliferation viability of MDA-MB-231 and SUM1315 cell lines, while downregulation of miR-195-5p could enhance growth ability (Figures 3A,B).

MDA-MB-231 and SUM1315 cell lines transfected with miR-195-5p mimics displayed a marked decreased in the percentage of EdU-positive cells, while knockdown revealed an opposite effect (Figure 3C).

As we all know, the role of CCNE1 is associated with regulating cell cycling. Thus, the effects of miR-195-5p in cell cycle distribution were tested by flow cytometry analysis. The increased percentage of G0–G1 phases were observed in high miR-195-5p-expressed MDA-MB-231 and SUM1315 cell lines, which also showed decreased percentage of S-phases. On the other hand, reduced miR-195-5p promoted G1-to-S-phase transformation (Figure 3D).

**Figure 4:**
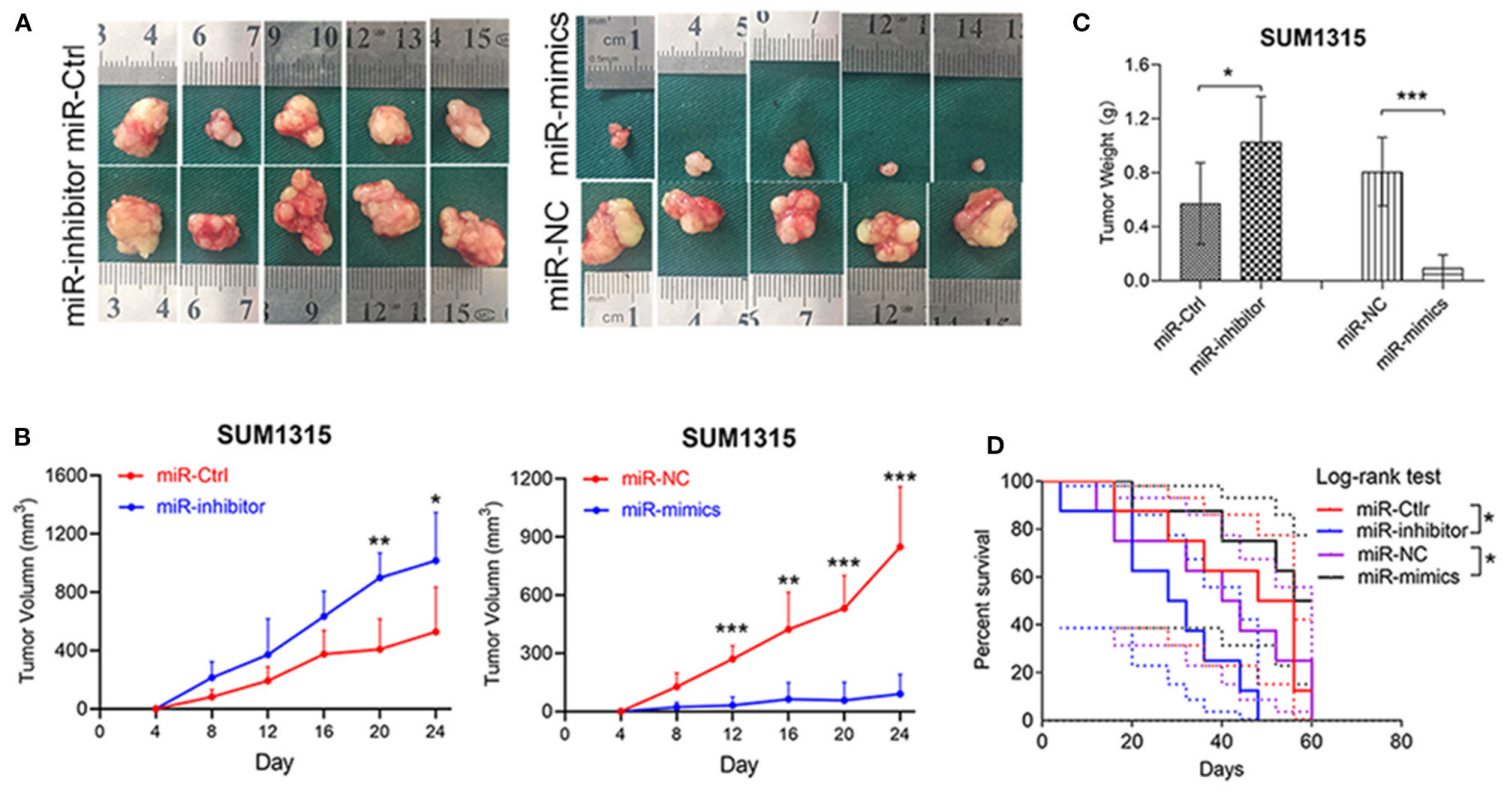
miR-195-5p facilitates tumorigenesis and development of TNBC cells *in vivo*. **(A)** Representative images of the subcutaneous tumors formed in nude mice following injection of transfected SUM1315 cells (*n* = 5 per group). **(B,C)** Tumor growth curves **(B)** and weight **(C)** are summarized. **(D)** Kaplan-Meier curves showed survival of four groups of nude mice (*n* = 8 per group) which started at 24 days after being injected. Data were presented as mean ± SD. *p < 0.05; **p < 0.01; ***p < 0.001.

The biological functions of miR-195-5p on tumor growth was also checked *in vivo* by a nude mice xenotransplantation model, in which SUM1315 cells stably transfected with miR-195-5p mimics, inhibitor, or respective negative control were injected subcutaneously. The results showed that tumor volume was remarkably increased in miR-195-5p inhibitor group compared with the negative control, while the difference of tumor weight was similar. Tumor growth curves were also utilized to depict tumor development in xenotransplantation model, and we found that tumor growth was significantly higher than control. In contrast, the opposite performance was presented in miR-195-5p mimics group (Figures 4A–C). Furthermore, miR-195-5p mimics injected model had better survival (Figure 4D).

Our results indicated that miR-195-5p downregulation in TNBC could promote tumor growth by mediating cell cycle G1-to-S-phase transformation and increasing DNA replication.

### LncRNA SENP3-EIF4A1 Functions as a Sponge for miR-195-5p

**Figure 5:**
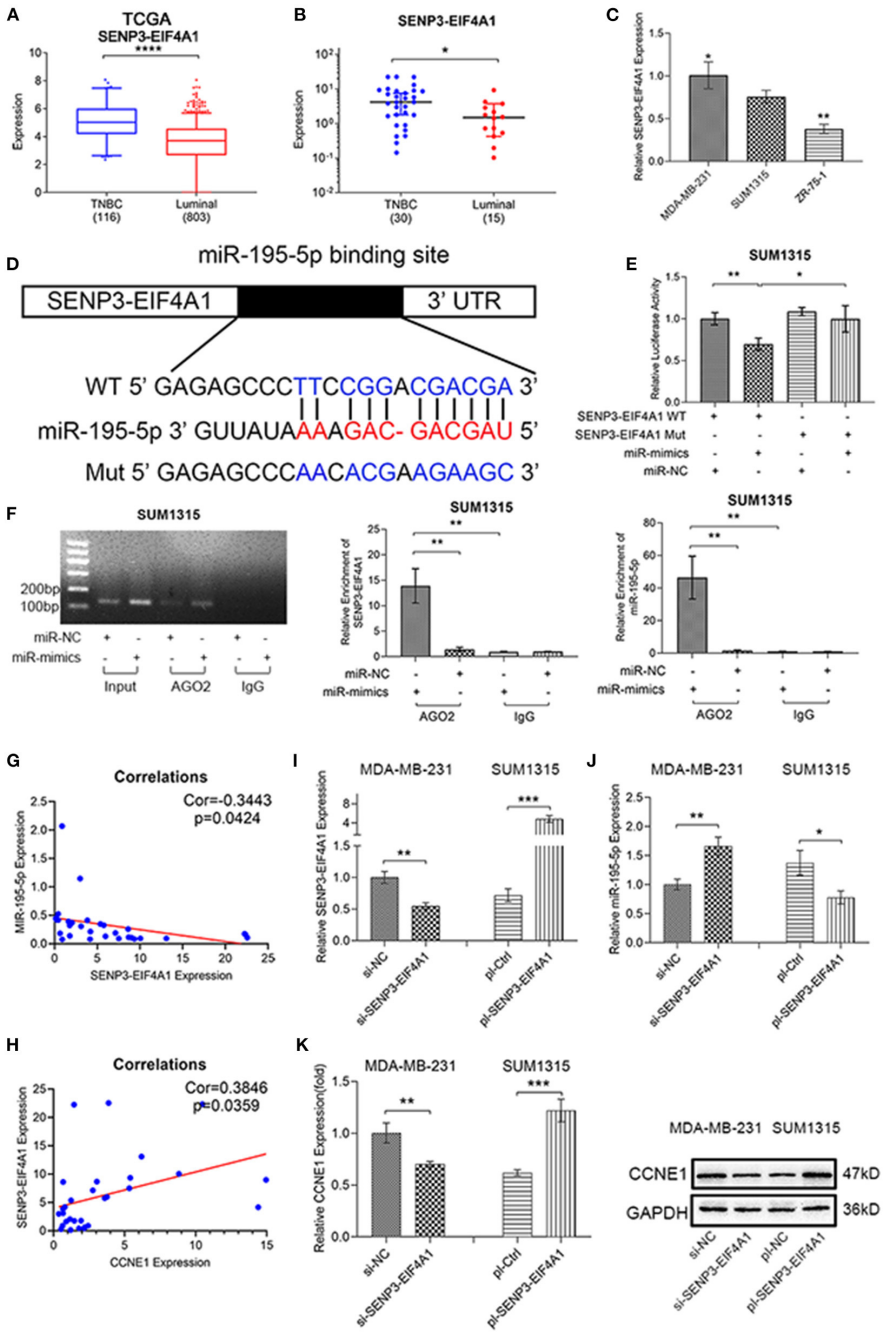
SENP3-EIF4A1 functions as a sponge for miR-195-5p. **(A–C)** SENP3-EIF4A1 expression in TNBC and luminal-type breast cancer from TCGA BRCA database **(A)**, clinical samples **(B)**, and cell lines **(C)**. **(D)** Schematic illustration of SENP3-EIF4A1 luciferase reporter vectors. **(E)** The relative luciferase activities were detected in SUM1315 cells after transfection. **(F)** Anti-AGO2 RIP was executed in transfected SUM1315 cells and agarose gel electrophoresis, qRT-PCR to detect expression. **(G,H)** Expression correlation between SENP3-EIF4A1 and miR-195-5p **(G)** or CCNE1 **(H)** in 30 TNBC samples. **(I,J)** SENP3-EIF4A1 **(I)** and miR-195-5p **(J)** expression shown by qRT-PCR after transfecting si-NC, si SENP3-EIF4A1, pl-Ctrl, and pl-SENP3-EIF4A1 in cells. **(K)** mRNA and protein level of CCNE1 expression of transfected cells. Data were presented as mean ± SD. *p < 0.05; **p < 0.01; ***p < 0.001.

MIRWALK were used to search for relative lncRNA, in order to define whether ceRNA plays an important role in anomalous miR-195-5p expression in TNBC or not. Then we found CCNE1 shares the highly similar MRE of miR-195-5p with SENP3-EIF4A1 (Figures 2I, 5D). Using the TCGA BRCA database and our clinical breast cancer data, SENP3-EIF4A1 presented higher expression in TNBC than luminal-type breast cancer samples (Figures 5A,B). Furthermore, in two TNBC cell lines MDA-MB-231 and SUM1315, SENP3-EIF4A1 expression was higher than ZR-75-1 cell line (a kind of luminal-type breast cancer cell) (Figure 5C). Interestingly, high level of SENP3-EIF4A1 expression in TNBC showed bad overall survival (OS), according to the TCGA BRCA database (Supplementary Figure 1E).

A brand new and relative dual-luciferase reporter assay was constructed (Figure 5D). The results showed the luciferase activity of miR-195-5p was notably reduced in SUM1315 cells with wild-type SENP3-EIF4A1 3′UTR, whereas the activity did not change with mutated SENP3-EIF4A1 3′UTR (Figure 5E). Generally acknowledged, miRNAs bind to Argonaute 2 (AGO2), which is a key part of RNA-induced silencing complex (RISC), and then regulate targets. Thus, the RIP assay was constructed in SUM1315 cells, while IgG as a negative control. Indeed, SENP3-EIF4A1 was remarkably enriched in miR-195-5p upregulated SUM1315 cells, compared with negative control group. Similarly, the level of miR-195-5p which enriched in miR-195-5p upregulated SUM1315 cells was high (Figure 5F).

In order to further understand the relationship of SENP3-EIF4A1, miR-195-5p, and CCNE1 expression in TNBC, Pearson correlation analysis was used in clinical breast cancer samples and TCGA BRCA database. The results demonstrated that SENP3-EIF4A1 was negatively correlated with miR-195-5p, while positively correlated with CCNE1 expression in TNBC (Figures 5G,H). Then, relevant plasmid vectors were designed to knockdown SENP3-EIF4A1 expression in MDA-MB-231 cells and raise in SUM1315 cells (Figure 5I). miR-195-5p expression was downregulated in SENP3-EIF4A1-reduced MDA-MB-231 cells and upregulated in SENP3-EIF4A1-overexpressed SUM1315 cells, compared with negative controls, respectively (Figure 5J). Besides, SENP3-EIF4A1 low-expressed MDA-MB-231 cells presented low mRNA and protein level of CCNE1 expression. On the contrary, SENP3-EIF4A1-overexpressed SUM1315 cells showed high expression of CCNE1 (Figure 5K).

Thus, we found that SENP3-EIF4A1 could function as a sponge for miR-195-5p and then regulate CCNE1 expression.

### DNA Methylation as Another Regulatory Factor of miR-195-5p

**Figure 6:**
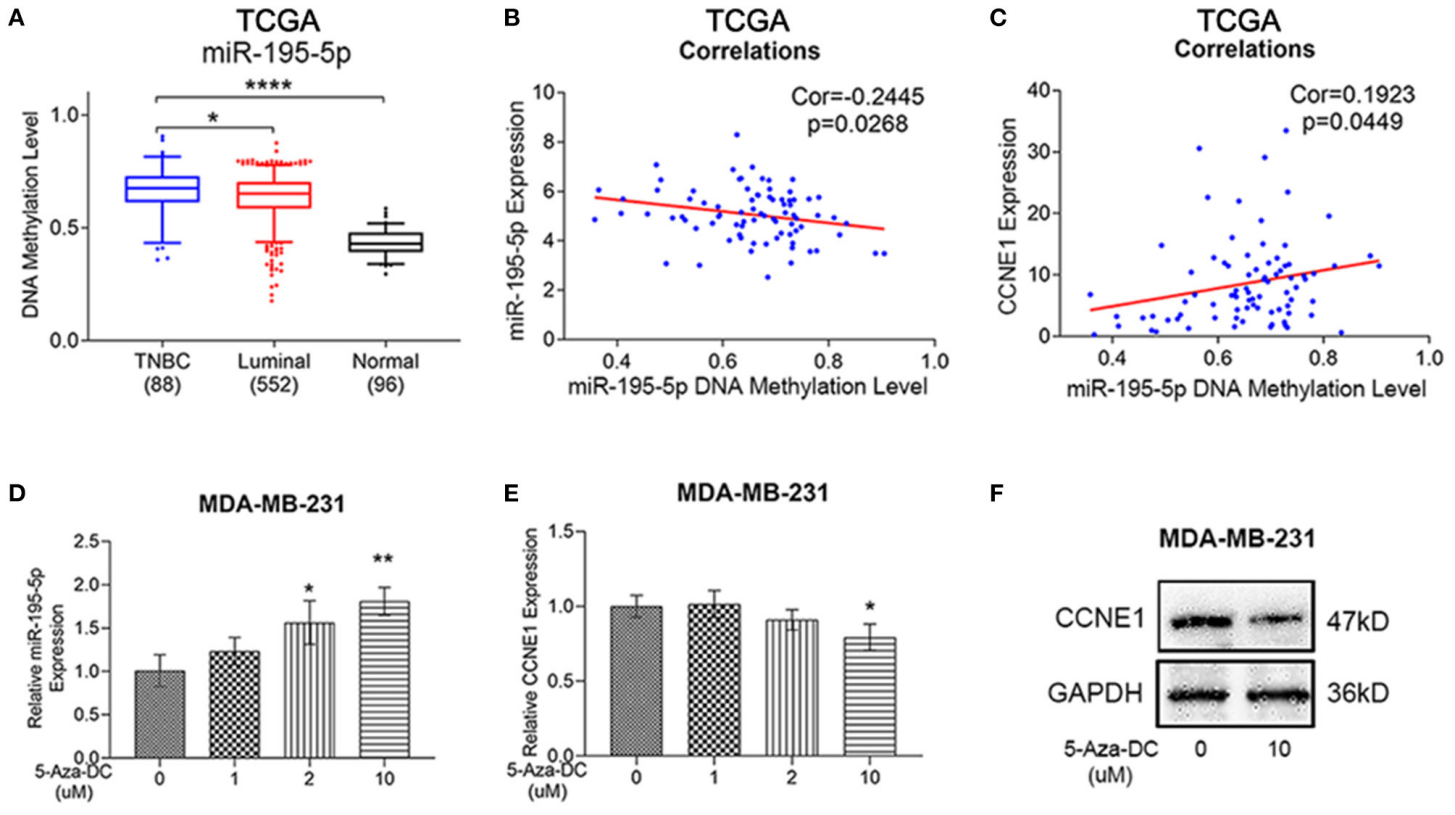
Methylation levels of miR-195-5p promoter region in TNBC. **(A–C)** TCGA BRCA database showed DNA methylation of miR-195-5p in TNBC samples as compared with luminal-type breast cancer or normal breast samples **(A)**, miR-195-5p DNA methylation level and miR-195-5p expression correlation in TNBC (*n* = 82) **(B)**, miR-195-5p DNA methylation level, and CCNE1 expression correlation in TNBC (*n* = 82) **(C)**. **(D)** qRT-PCR of miR-195-5p expression in MDA-MB-231 cells with or without 5-AzaDc treatment. **(E,F)** mRNA **(E)** and protein **(F)** level of CCNE1 expression in MDA-MB-231 cells with or without treatment. *p < 0.05; **p < 0.01; ****p < 0.0001.

Using the TCGA BRCA methylation database, we obtained additional discovery that DNA methylation of miR-195-5p promoter was higher in TNBC than luminal-type breast cancer and normal samples (Figure 6A). Moreover, the level of miR-195-5p DNA methylation was negatively correlated with miR-195-5p expression, while positively correlated with CCNE1 expression in TNBC, according to the TCGA BRCA database (Figures 6B,C). To verify whether DNA methylation plays a role in expression of miR-195-5p, different doses of DNA methyltransferase inhibitor 5-Aza-DC was utilized to co-culture with MDA-MB-231 cells. With the application of 5-Aza-DC, MDA-MB-231 expressed higher miR-195-5p and lower CCNE1 levels (Figures 6D–F).

Thus, we found that SENP3-EIF4A1 could function as a sponge for miR-195-5p and then regulate CCNE1 expression.

### SENP3-EIF4A1 Promotes TNBC Cell Proliferation Through SENP3-EIF4A1/miR-195-5p/CCNE1 Axis

**Figure 7:**
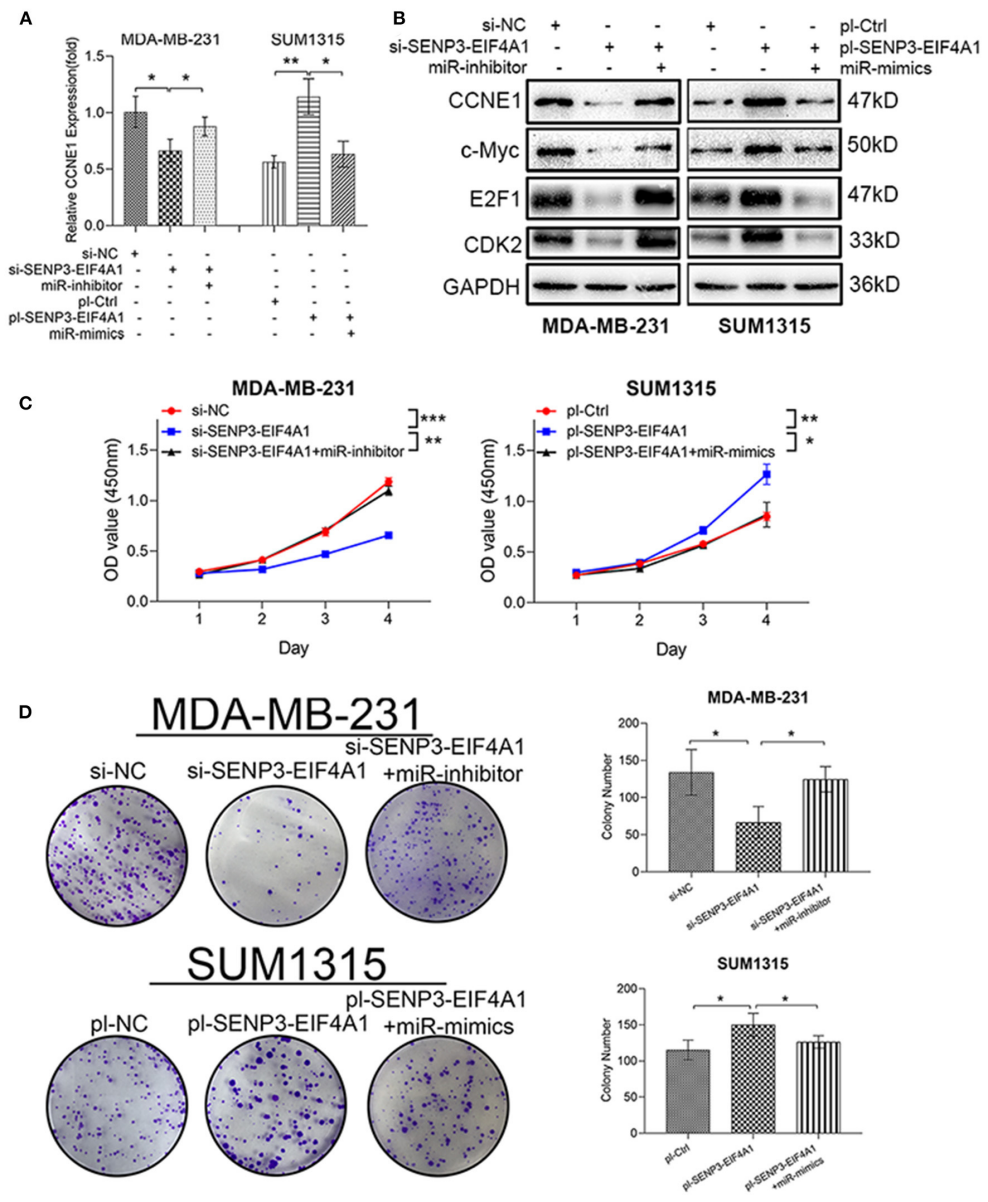
SENP3-EIF4A1 promotes cell growth through SENP3-EIF4A1/miR-195-5p/CCNE1 axis. **(A)** CCNE1 mRNA expression of transfected cells. **(B)** CCNE1 and related c-Myc, E2F1, and CDK2 protein expression of transfected cells. **(C)** The proliferation curves of transfected cells were evaluated by CCK8 assays. **(D)** Colony formation assays were used to detect the growth of transfected cells. Data were presented as mean ± SD. *p < 0.05; **p < 0.01; ***p < 0.001.

To validate whether circAGFG1 and miR-195-5p could regulate the expression of CCNE1 cooperatively in TNBC cells, we found that the down- or upregulation of CCNE1 mediated by SENP3-EIF4A1 knockdown or overexpression could be reversed by miR-195-5p inhibitor or mimics, respectively (Figures 7A,B). c-Myc, E2F1, and CDK2 were important members of CCNE1 function pathways. We further found c-Myc, E2F1, CDK2, and CCNE1 expression presented highly positive correlation in TNBC, according to the TCGA BRCA database (Supplementary Figure 1H). Interestingly, similar to changes of CCNE1 expression, the protein expression of c-Myc, E2F1 and CDK2 were also reduced or upregulated by SENP3-EIF4A1 knockdown or overexpression, and these effects could be neutralized using miR-195-5p (Figure 7B).

CCK8 and colony formation assays were performed to test cell-proliferation function of SENP3-EIF4A1 in TNBC. We found that downregulation of SENP3-EIF4A1 could reduce proliferation viability of MDA-MB-231 cells, while upregulation of SENP3-EIF4A1 could enhance proliferative effects in SUM1315 cells. Furthermore, the functions of SENP3-EIF4A1 in these two TNBC cells could be neutralized by knockdown or overexpression of miR-195-5p (Figures 7C,D).

## Discussion

TNBC is a breast cancer subtype with high degree of malignancy and heterogeneity. Due to poor prognosis and limited treatment, TNBC has become one of the cruelest killers of female health. Now, it is urgent to discover novel targets for accurate diagnosis methods and effective treatment strategy. As important cell cycle regulators, cyclins participate in a variety of physiological activities with CDKs and CKIs. Dysregulation (Qie and Diehl, 2020) or even mislocalization (Moore, 2013) of cyclins could promote tumorigenesis in several cancers, including breast cancer (Dong et al., 2014; Ju et al., 2019). In order to better understand the effect of different cyclins in breast cancer, we analyzed their expression through the TCGA BRCA database and selected CCNE1, which was highly expressed in tumor, as further research object. A pan-cancer analysis based on TCGA and GTEx databases demonstrated CCNE1 was prominently expressed in nearly all human cancers. High expression of CCNE1 in breast cancer (Sieuwerts et al., 2006), liver cancer (Aziz et al., 2019), and ovarian cancer (Au-Yeung et al., 2017) indicates poor prognosis. Analyzing TCGA BRCA clinical data, we further found that CCNE1 expression in breast cancer could be related to TNM stage and ER/PR status. Moreover, the TCGA BRCA data showed CCNE1 was overexpressed in TNBC than luminal-type breast cancer and normal samples, while high CCNE1 expression in TNBC meant bad survival. Clinical samples and breast cancer cell lines also confirmed the expression of CCNE1. The mechanism of CCNE1 expression dysregulation in TNBC should be explored.

MicroRNA, a non-encoding RNA of 18–25 nucleotides, is considered a posttranscriptional regulator of target mRNA by microRNA response element (Garzon et al., 2010; Kasinski and Slack, 2011). Studies have shown that microRNA such as miR-204 (Yang et al., 2018), miR-375 (Tang et al., 2019), and miR-3178 (Kong et al., 2018) could play important roles in the occurrence and development of TNBC, while microRNA could also participate in the regulation of CCNE1 in tumor (Li et al., 2019). Thus, we applied microRNA forecasting tools to screen correlative microRNA and selected miR-195-5p, which downregulated colorectal cancer and affected tumorigenicity and metastasis (Lin et al., 2019). Our study revealed that miR-195-5p expression was relatively low in TNBC, which had significantly negative correlations with CCNE1 expression. Furthermore, highly expressed miR-195-5p in TNBC meant good prognosis. After constructing miR-mimics/inhibitor-TNBC cell lines, dual-luciferase reporter assay and expression of CCNE1 mRNA or protein level demonstrated that miR-195-5p was the potential direct up-stream factor of CCNE1. The results of cell proliferation, cell cycle assay, and xenotransplantation model animal experiment depicted that loss of mir-195-5p could promote tumor growth and induce poor prognosis, which might attribute to cell cycle G1-to-S-phase transformation and DNA replication increase.

lncRNA-microRNA-associated ceRNA has been confirmed as an important mode in progress of TNBC (Yang et al., 2018; Dong et al., 2020; Li et al., 2020), and CCNE1-associated ceRNA regulation appeared in several cancers (Zhang et al., 2019, 2020). Thus, we inferred that lncRNA-microRNA-associated ceRNA hypothesis provided potential possibility of CCNE1 dysregulation in TNBC. Forecasting tools recommended SENP3-EIF4A1 as a candidate, which is almost unknown to us except the participating ceRNA regulation in hepatocellular carcinoma (Wang J. et al., 2020). Then we performed experiments to confirm that SENP3-EIF4A1 expression was higher in TNBC than luminal-type breast cancer, which had significantly negative correlations with miR-195-5p expression and positive correlations with CCNE1 expression. Moreover, high expression of SENP3-EIF4A1 in TNBC meant poor prognosis. RIP and dual-luciferase reporter assay indicated that SENP3-EIF4A1 could directly combine with miR-195-5p by a similar MRE to CCNE1 combination. Immediately, SENP3-EIF4A1 showed abilities of regulating miR-195-5p expression and CCNE1 mRNA and protein expression in TNBC. Performing cell proliferation assays and promoting cell growth functions of SENP3-EIF4A1 were ensured, and these could be blocked by overexpression of miR-195-5p in TNBC cells. Deeply, miR-195-5p upregulation in TNBC could be capable of rescuing protein expression of CCNE1 and closely related c-Myc, CDK2, and E2F1 upregulated by SENP3-EIF4A1. CKD2, a kind of CKDs, is composed of CCNE1-CKD2 complex and participates in the regulation of cell cycle activity (Mende et al., 2015), then released E2F1, which is recognized as an oncogene (Rodriguez-Bravo et al., 2018; Liu et al., 2019). MYC is another well-known oncogene, which could be activated by E2F1 in tumor (Wang et al., 2014). Thus, we found that SENP3-EIF4A1 could function as a sponge for miR-195-5p and then regulate CCNE1 expression.

Interestingly, DNA methylation level of miR-195-5p was abnormally high in TNBC according to the TCGA database and had negative correlations with miR-195-5p expression and positive correlations with CCNE1. Then, using 5-Aza-DC could increase miR-195-5p and decrease CCNE1 expression in TNBC cells. Abnormal methylation is an important way to promote the development of TNBC cells (Chen et al., 2019). It has been found that DNA hyper/hypomethylation is a potential mechanism of miRNA dysregulation in breast cancer (Aure et al., 2013; Kang et al., 2016). Thus, DNA hypermethylation of miR-195-5p promotor might enhance CCNE1 expression in TNBC synergistically.

**Figure 8:**
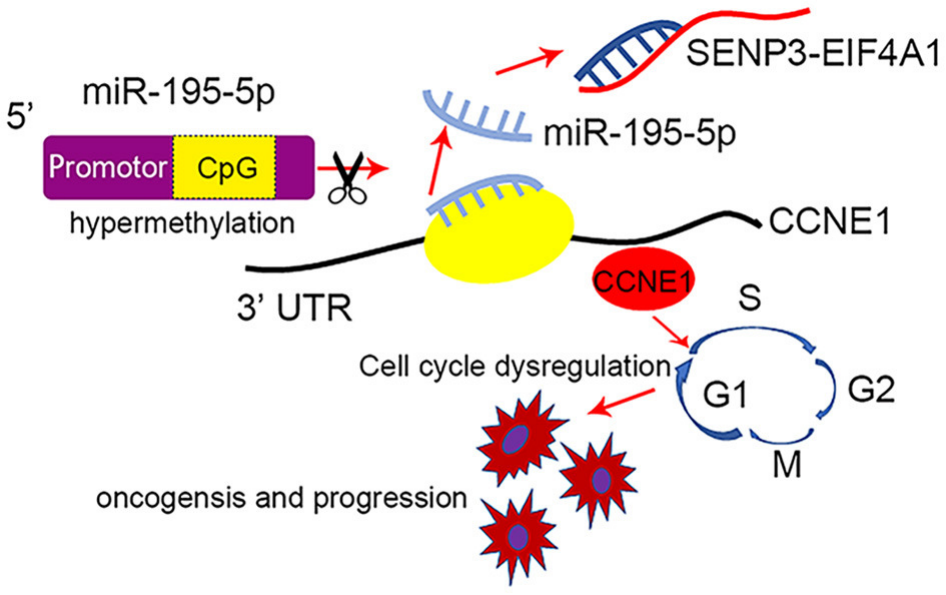
Schematic model of SENP3-EIF4A1/miR-195-5p/CCNE1 axis in regulating TNBC. DNA hypermethylation of miR-195-5p decreases miR-195-5p and the lncRNA SENP3-EIF4A1 functions as a sponge of miR-195-5p to promote TNBC development through increasing CCNE1 expression.

In conclusion, the lncRNA SENP3-EIF4A1 functions as a sponge of miR-195-5p to promote TNBC development through increasing CCNE1 expression, while DNA hypermethylation of miR-195-5p may be another reason for CCNE1 dysregulation in TNBC (Figure 8). Generally, our findings of this study will help to explore the brand-new mechanism of ceRNA in the occurrence and progression of TNBC and contribute to novel diagnosis and treatment.

## Data Availability Statement

Publicly available datasets were analyzed in this study. These data can be found here: TCGA (https://portal.gdc.cancer.gov/).

## Ethics Statement

The studies involving human participants were reviewed and approved by Nanjing Medical University Institutional Ethics Committee. The patients/participants provided their written informed consent to participate in this study. The animal study was reviewed and approved by Nanjing Medical University Institutional Animal Care and Use Committee.

## Consent for Publication

All authors give consent for the publication of the manuscript in this journal.

## Author Contributions

LC, XM, and CS performed the experiments. AQ and YZ analyzed the data. CC and ZL collected clinical data. XL and TW designed the experiments. All authors were involved in writing the paper and approved the submission and publication.

## Conflict of Interest

The authors declare that the research was conducted in the absence of any commercial or financial relationships that could be construed as a potential conflict of interest.

## Abbreviations

TNBC: triple-negative breast cancer
CCNE1: cyclin E1
miR: microRNA
ceRNA: competing endogenous RNA
LncRNA: long noncoding RNA
OS: overall survival
TCGA: The Cancer Genome Atlas
ER: estrogen receptor
PR: progesterone receptor
HER2: human epidermal growth factor receptor 2
qRT-PCR: real-time quantitative polymerase chain reaction
3 ′ UTR: 3 ′ -untranslated region
RIP: RNA immunoprecipitation.
