# Supplementary material for: Long Non-coding RNA SENP3-EIF4A1 Functions as a Sponge of miR-195-5p to Drive Triple-Negative Breast Cancer Progress by Overexpressing CCNE1
Source: Front Cell Dev Biol. 2021 Mar 15;9:647527. doi: 10.3389/fcell.2021.647527 (PMC8006396; doi:10.3389/fcell.2021.647527)
Supplement: Supplementary file 1 [file Table_1.doc]

**Table. S1 The primers for the targets in the study**

| CCNE1 | FW: 5’-GCCAGCCTTGGGACAATAATG-3’ |
| --- | --- |
| CCNE1 | RV: 5’-CTTGCACGTTGAGTTTGGGT-3’ |
| SENP3-EIF4A1 | FW: 5’ -CCGCCAGTTCTACATCAACG 3’ |
| SENP3-EIF4A1 | RV: 5’-TTCCTCCGGGTGTTGATGAA 3’ |
| GAPDH | FW: 5’-AGAAGGCTGGGGCTCATTTG-3’ |
| GAPDH | RV: 5’-AGGGGCCATCCACAGTCTTC -3’ |
| miR-195-5p | FW: 5’-ACACTCCAGCTGGGTAGCAGCACAGAAAT-3’ |
| miR-195-5p | RV: 5’-TGGTGTCGTGGAGTCG-3’ |
| U6 | FW: 5’-CTCGCTTCGGCAGCACA-3’ |
| U6 | RV: 5’-AACGCTTCACGAATTTGCGT-3’ |
