# Supplementary material for: Long Non-coding RNA SENP3-EIF4A1 Functions as a Sponge of miR-195-5p to Drive Triple-Negative Breast Cancer Progress by Overexpressing CCNE1
Source: Front Cell Dev Biol. 2021 Mar 15;9:647527. doi: 10.3389/fcell.2021.647527 (PMC8006396; doi:10.3389/fcell.2021.647527)
Supplement: Supplementary file 2 [file Image_1.pdf]

Fig. S1

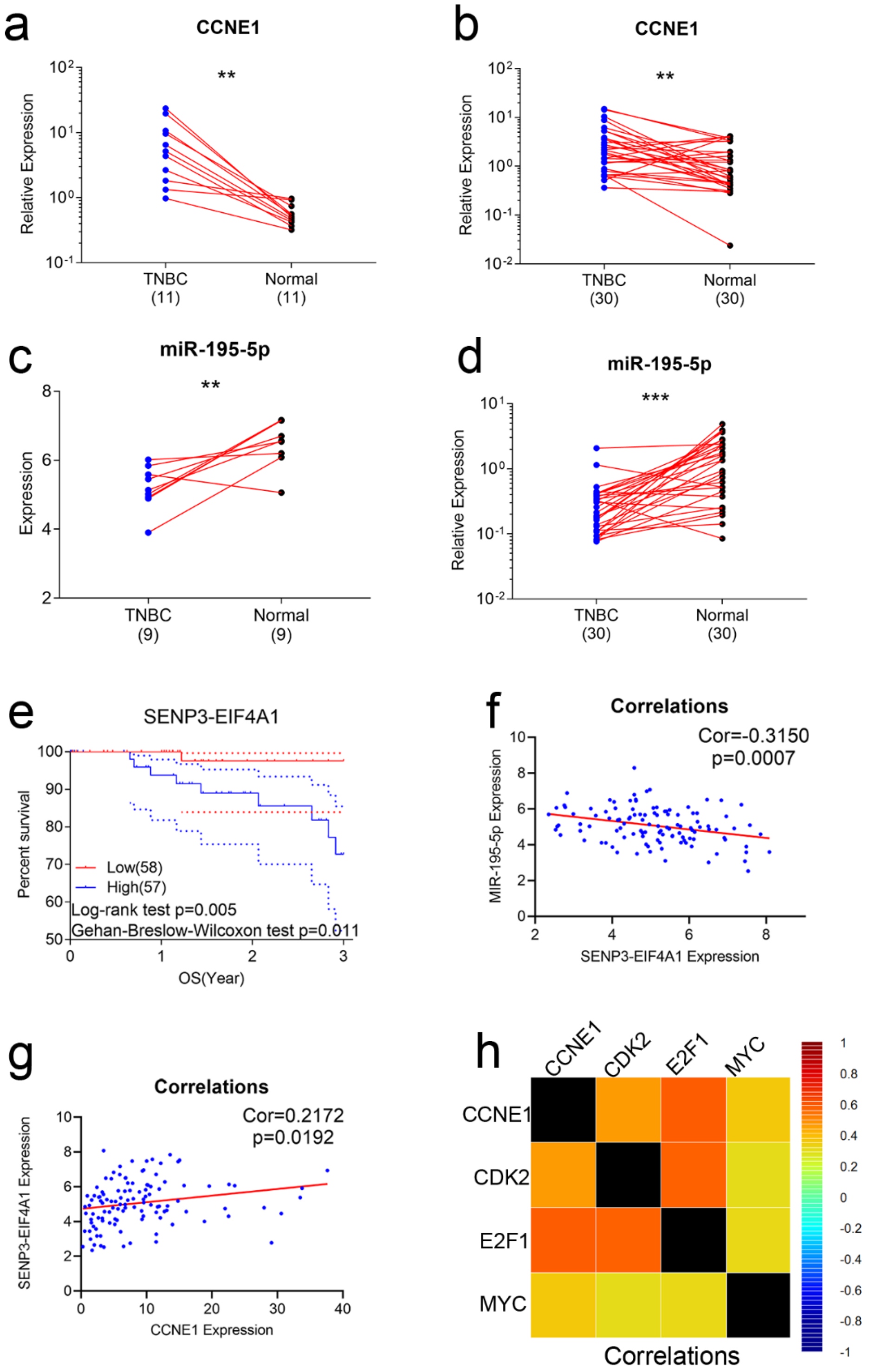

Fig. S1 (a and b) CCNE1expression in TNBC and paired normal breast samples from TCGA BRCA database(n=11) (a) and clinical samples(n=30) (b). (c and d) CCNE1expression in TNBC and paired normal breast samples from TCGA BRCA database(n=9) (c) and clinical samples(n=30) (d). (e) SENP3-EIF4A1 expression related 3-year overall survival (n=115) in TNBC. (f) SENP3-EIF4A1 and miR-195-5p expression correlations in TNBC from TCGA (N=113). (g) SENP3-EIF4A1 and CCNE1 expression correlations in TNBC from TCGA (N=116). (h) CCNE1 and related CKD2, E2F1 and c-Myc expression correlations in TNBC from TCGA.
